# The Identification and Validation of a Robust Immune-Associated Gene Signature in Cutaneous Melanoma

**DOI:** 10.1155/2021/6686284

**Published:** 2021-02-19

**Authors:** Le-Bin Song, Jiao-Chen Luan, Qi-Jie Zhang, Lin Chen, Hao-Yang Wang, Xue-Chen Cao, Ning-Hong Song, Yan Lu

**Affiliations:** ^1^Department of Dermatology, The First Affiliated Hospital of Nanjing Medical University, Nanjing, China; ^2^Department of Urology, The First Affiliated Hospital of Nanjing Medical University, Nanjing, China; ^3^The Affiliated Kezhou People's Hospital of Nanjing Medical University, Kezhou, Xinjiang, China

## Abstract

**Background:**

Cutaneous melanoma is defined as one of the most aggressive skin tumors in the world. An increasing body of evidence suggested an indispensable association between immune-associated gene (IAG) signature and melanoma. This article is aimed at formulating an IAG signature to estimate prognosis of melanoma.

**Methods:**

434 melanoma patients were extracted from The Cancer Genome Atlas (TCGA) database, and 1811 IAGs were downloaded from the ImmPort database in our retrospective study. The Cox regression analysis and LASSO regression analysis were utilized to establish a prognostic IAG signature. The Kaplan-Meier (KM) survival analysis was performed, and the time-dependent receiver operating characteristic curve (ROC) analysis was further applied to assess the predictive value. Besides, the propensity score algorithm was utilized to balance the confounding clinical factors between the high- and low-risk groups.

**Results:**

A total of six prognostic IAGs comprising of INHA, NDRG1, IFITM1, LHB, GBP2, and CCL8 were eventually filtered out. According to the KM survival analysis, the results displayed a shorter overall survival (OS) in the high-risk group compared to the low-risk group. In the multivariate Cox model, the gene signature was testified as a remarkable prognostic factor (HR = 45.423, *P* < 0.001). Additionally, the ROC curve analyses were performed which demonstrated our IAG signature was superior to four known biomarkers mentioned in the study. Moreover, the IAG signature was significantly related to immunotherapy-related biomarkers.

**Conclusion:**

Our study demonstrated that the six IAG signature played a critical role in the prognosis and immunotherapy of melanoma, which might help clinicians predict patients' survival and provide individualized treatment.

## 1. Introduction

Cutaneous melanoma is an aggressive malignancy associated with significant clinical, biological, and epidemiological heterogeneity [1]. The incidence of cutaneous melanoma patients in Asians was 1.5 per 100,000 [2]. Although cutaneous melanoma occupies about 3 percent of the overall skin cancers, it is responsible for a tremendous shocking number of deaths (65 percent) [3]. Besides, the 5-year survival rate of advanced metastatic melanoma is approximately up to 10~15 percent due to its poor prognosis [4]. As for the pathogenesis of melanoma, the exposure to ultraviolet rays is regarded as the most significant and probable environmental risk factor in cutaneous melanoma due to its effect of genotoxicity [5]. The number of melanocytic nevi, family history, and hereditary susceptibility are also identified as the most indispensable host risk factors in cutaneous melanoma [6]. Different from other malignancies, early detection and early treatment in cutaneous melanoma are related with a favorable survival rate. Therefore, early diagnosis and proper therapies are particularly crucial for melanoma [7].

Currently, the treatment options for melanoma are determined by the stage of the cancer and the location of the melanoma [8]. Surgical resection is the ideal treatment for melanoma, including intraoperative lymph node localization or regional selective lymph node resection [9]. Chemotherapy or combination chemotherapy can also be utilized in metastatic patients [10]. However, late and progressive stages of melanoma are usually resulting in poor prognosis. The immune system has been shown to be a determinative factor during cancer initiation and progression [11, 12]. Hence, immune-based therapy has recently approved the dramatical and innovative efficiency for the treatment of advanced and metastatic cutaneous melanoma [13]. Various evidence has proved that cutaneous melanomas are immune-related tumors, and immunotherapy is vigorously pursued through targeting the immune checkpoints [14, 15]. Several of molecules have been described and were identified as potential targets in immunotherapy: the cytotoxic T-lymphocyte antigen (CTLA-4), the programmed cell death protein 1 (PD-1), the programmed cell death-ligand 1 (PD-L1), the killer cell immunoglobulin-like receptor (KIR), the lymphocyte-activation gene 3 (LAG3), and the T cell immunoglobulin domain and mucin domain-3 (TIM3) [16]. The programmed cell death-ligand 1 (PD-L1) and the programmed cell death-ligand 2 (PD-L2) are primarily expressed in the tumor microenvironment and can lead to inhibition of T cell stimulation after ligation to PD-1. Now, A growing number of studies that are related to immune-based gene prognostic biomarkers for tumors are ongoing and may improve the accuracy of immunotherapy. Therefore, the immune-related prognostic signature may play crucial roles in cutaneous melanoma.

In this manuscript, we analyzed immune-related genes from large amounts of cutaneous melanoma transcriptional data. A prognostic immune-associated gene (IAG) signature was constructed by a combination of multiple immune genes. Moreover, the prognostic prediction value of the IAGS was systematically validated, which would be conducive to formulate the therapeutic schedule for patients with cutaneous melanoma. Additionally, we investigated the relationship between the IAG signature and some other immunotherapy-related biomarkers.

## 2. Materials and Methods

### 2.1. Data Collection

RNA-sequencing (RNA-seq) expression profile and related clinical follow-up parameters of melanoma cohorts were downloaded from The Cancer Genome Atlas (TCGA) RNA-seq in our retrospective study. Overall, 434 melanoma patients were enrolled in our research, and specific clinicopathological characteristics are shown in Table 1. The patients were further randomly assigned to a training set and a testing set by a ratio of 2 to 1. The comprehensive list of immune related-genes was extracted from The Immunology Database and Analysis Portal (ImmPort, https://immport.niaid.nih.gov), containing a total of 1811 immune-associated genes (IAGs). The comprehensive list of immune related-genes was extracted from The Immunology Database and Analysis Portal (ImmPort, https://immport.niaid.nih.gov), containing a total of 1811 immune-associated genes (IAGs). The richness of T cell receptor/B cell receptor (TCR/BCR) was obtained from Thorsson et al. [16] (https://gdc.cancer.gov/about-data/). Immune checkpoint genes were acquired from Auslander et al. [17] CYT (cytolytic activity) was computed as the geometric mean of the gene expression of two cytolytic markers (PRF1 and GZMA) [18]. In addition, based on the T cell-inflamed gene expression profile (GEP) gene signature from Ayers et al. [19] and the gene signatures of six immune cell populations from Charoentong et al. [20], we calculated the relative abundance of GEP level and the immune cell population in each patient by utilizing gene set variation analysis (GSVA) [21].

### 2.2. Identification and Establishment of the IAG Signature Risk Model

The univariate Cox regression analysis was carried out to estimate the relationship between each IAG and melanoma patients' overall survival in the training database. We chose *P* = 0.05 as a cut-off for the correlation analysis, and the eligible genes were identified for prognostic signature development. Then, we adopted a least absolute shrinkage and selection operator (LASSO) Cox regression method to single out the optimal prognostic IAGs and construct a multigene signature associated with IAGs. LASSO regression could fit the generalized linear model that led to variable selection and regularization. Additionally, overfitting to a certain degree was avoided, because the complexity of LASSO regression was controlled by the coefficient (*γ*), and a less variable model was obtained by utilizing a penalty ratio in terms of their size. Based on the multiple analyses above, our candidate prognostic IAGs were finally screened out to establish the risk score model. Subsequently, in accordance with the regression coefficients calculated by the LASSO regression model, the risk score algorithm of each melanoma patient for predicting OS was constructed as follows:

(1) $$\begin{array}{c} \text{Risk score}=\overset{n}{\underset{i=1}{\sum }}\exp i*\beta i. \end{array}$$

Specifically, where *β*_*i*_ is the regression coefficient of gene *i*, exp*i* is the expression value of each prognostic gene *i*, and *n* is the number of candidate genes. We calculated the risk score of each melanoma patient by a linear combination of the expression value of candidate genes weighted by the regression coefficient (*β*).

### 2.3. Verification and Assessment of the IAG Signature

Based on the median risk score in the training cohort as the threshold, all eligible melanoma patients were stratified into the high- and low-risk groups in the training database and testing dataset for the following analysis. The KM survival analysis was employed to compare the prognostic difference in survival status between the high- and low-risk subgroups, and the time-dependent receiver operating characteristic curve (ROC) analysis was applied to validate and assess the predictive accuracy of the IAG signature in both databases. Moreover, under the univariate and multivariate Cox's proportional hazard regression model, we estimated whether the six IAG signature was independent of other clinicopathological parameters.

### 2.4. Propensity Score Algorithm

The propensity score algorithm was used to balance confounding parameters, such as age, gender, Breslow depth, Clark level, T stage, N stage, M stage, tumor stage, and anatomic site between the high-risk and low-risk groups [22]. We used the nearest neighbor matching within a caliper of 0.05 on the propensity score with a matching ratio of 1 : 1 [23]. Then, the covariate balance was checked to assess the adequacy of the propensity model. We finally compared the immune-related features between the high-risk and low-risk groups and considered *P* < 0.05 as significant.

### 2.5. Statistical Analysis

Student's *t*-test or Wilcoxon rank-sum test was performed for continuous variables and chi-squared or Fisher's exact test for categorical variables. The Cox regression analyses and Pearson correlation analysis were utilized to infer the candidate immune-related genes. Hazard ratios (HR) and related 95% confidence interval (CI) were evaluated by the Cox proportional hazard models. The Kaplan-Meier survival curves were plotted by the “survival” package to indicate patients' cumulative survival time at risk for several time points, and the ROC analysis was conducted by the “timeROC” package, and the area under curve (AUC) along with 95% CI was correspondingly obtained to predict OS of patients. All statistical analyses were carried out by utilizing R software 3.6.3 (R Foundation for Statistical Computing, Vienna, Austria) and IBM SPSS Statistics 22.0 (SPSS Inc.). Two-tailed *P* values < 0.05 were considered statistically significant for the whole statistical analyses.

## 3. Results

### 3.1. Clinicopathological Features of the Study Samples

Totally, 434 melanoma patients with clinical and pathological parameters from the TCGA dataset were included in our study. In the light of TCGA series number, all samples were randomly divided into two datasets: the first two-thirds were used as the training set (*n* = 289) to identify and establish the IAG signature, and the remaining one-third were utilized as the testing set (*n* = 145) to verify and assess the accuracy of the prognostic biomarker in predicting patients' OS. The detailed distribution and selected demographic characteristics are shown in Table 1.

### 3.2. Construction and Verification of the Prognostic IAG Signature Risk Score

**Figure 1:**
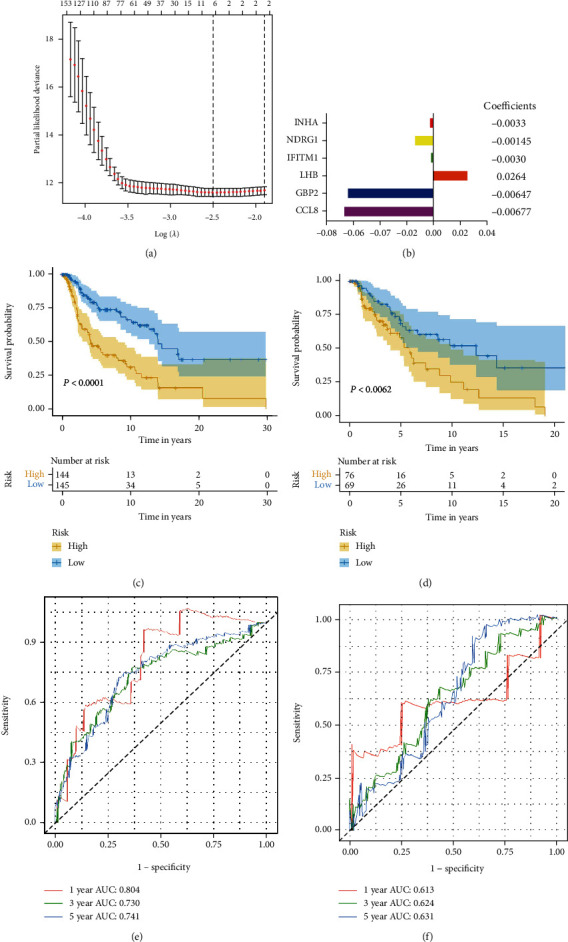
Correlation between the six IAGs and overall survival of cutaneous melanoma. (a) The least absolute shrinkage and selection operator (LASSO) Cox regression method. (b) The regression coefficient of the optimal prognostic IAGs. (c) Kaplan-Meier curves of overall survival of the high-risk and low-risk groups in (c) the training set and (d) the testing set. The ROC curves of 1-year, 3-year and 5-year OS in (e) the training set and (f) the testing set. IAGs: immune-associated genes; OS: overall survival; ROC: receiver operating characteristic.

A total of 1811 IAGs were downloaded from the ImmPort database. To investigate the relationship between the overall survival (OS) and IAGs in the training set, the univariate Cox's regression analysis was performed. *P* = 0.05 was considered a cut-off for the correlation analysis, and we narrowed down 423 eligible IAGs for prognostic risk score development. Subsequently, based on the LASSO Cox analysis, six optimal prognostic IAGs comprising of INHA, NDRG1, IFITM1, LHB, GBP2, and CCL8 were screened out (Figures 1(a) and 1(b)). Next, based on the regression coefficients and the expression value of candidate genes, we constructed the prognostic IAG signature. The risk score of each patient was calculated in accordance with the following formula: risk score = (−0.0033∗expression value of INHA) + (−0.0145∗expression value of NDRG1) + (−0.0030∗expression value of IFITM1) + (0.0264∗expression value of LHB) + (−0.0647∗expression value of GBP2) + (−0.0677∗expression value of CCL8). Moreover, Kaplan-Meier survival analysis was established to assess the predictive ability of the risk score, and 289 samples in the training set were divided into the high-risk (*n* = 144) and low-risk (*n* = 145) groups according to the median value. Kaplan-Meier survival curves displayed that the melanoma patients had better OS compared to those in the high-risk one (*P* < 0.0001) (Figure 1(c)). To further estimate the prognostic veracity of the risk score, we applied the time-dependent ROC curve, and the area under the curve (AUC) values of 1-year, 3-year, and 5-year OS were 0.804, 0.730, and 0.741, respectively (Figure 1(e)). The results displayed superior predictive accuracy in patients' survival.

In addition, to verify the prognostic value of the IAG signature risk score, we utilized the testing set (*n* = 145) in light of the same algorithm and regression coefficient (*β*) from the training dataset. Based on the median risk score in the training set, 145 melanoma patients were divided in the high-risk group (*n* = 76) and low-risk (*n* = 69) group. Regarding the Kaplan-Meier survival analysis, the testing set showed similar outcomes (*P* = 0.0062) as expected (Figure 1(d)). Time-dependent ROC analysis of the risk score model demonstrated that AUC for 1-year, 3-year, and 5-year OS of the testing dataset were 0.613, 0.624, and 0.631, respectively (Figure 1(f)). In all, the prognostic IAG signature had better sensitivity and specificity in predicting the OS of melanoma patients.

### 3.3. IAG Signature Was Critically Associated with Clinicopathological Characteristics

**Figure 2:**
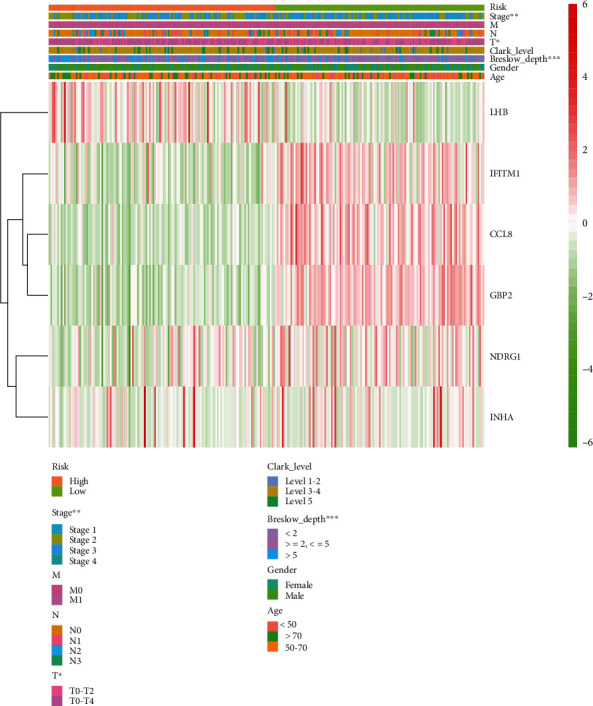
The heat map that showed the expression of six IAGs and the distribution of clinicopathological variables. IAGs: immune-associated genes.

A heat map was plotted to reflect the potential relationship between the IAG signature and several clinicopathological characteristics in the high and low-risk groups. The clinical pathological features presented distributed patterns corresponding to the risk score. We found that T stage (*P* < 0.05), tumor stage (*P* < 0.01), and Breslow depth (*P* < 0.001) were significantly concerned with the signature. Besides, we also plotted the heat map to exhibit the expression value of six different IAGs between the high- and low-risk groups (Figure 2).

### 3.4. Independent Risk Parameter Analysis

**Figure 3:**
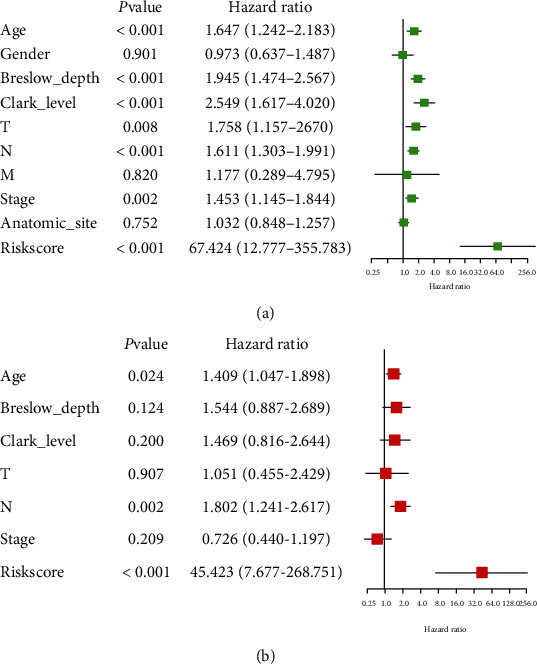
The univariate and multivariate Cox regression analyses of clinicopathological factors (including the risk score) and OS in the TCGA database. OS: overall survival. ^∗^*P* < 0.05, ^∗∗^*P* < 0.01, and ^∗∗∗^*P* < 0.001.

Univariate and multivariate Cox's regression models were employed to estimate the independent risk parameters in the whole TCGA set. The IAG signature and clinical factors (age, gender, Breslow depth, Clark level, T, N, M, stage, and anatomic site) were included. The univariate regression analysis revealed that age (*P* < 0.001), Breslow depth (*P* < 0.001), Clark level (*P* < 0.001), N stage (*P* < 0.001), risk score (*P* < 0.001), T stage (*P* = 0.008), and tumor stage (*P* = 0.002) were significantly related to the melanoma patients' prognosis, while gender, M stage, and anatomic site had no strong association with overall survival (*P* > 0.05) (Figure 3(a)). Multivariate regression analysis demonstrated that risk score (*P* < 0.001), age (*P* = 0.024), and N stage (*P* = 0.002) had a remarkable prognostic value compared with other clinicopathological parameters in the TCGA set (Figure 3(b)). All in all, these results showed that our IAG signature proved to be an independent prognostic factor in melanoma.

### 3.5. Stratification Analysis

In our study, we further decided to stratify the patients in each database into subgroups comprising of age, gender, anatomic site, Breslow depth, Clark level, stage, pathologic TNM, and tumor location, and each subgroup was further distributed into the high- and low-risk groups. In the Breslow depth (<2 mm), anatomic site (head and neck or extremities or trunk), pathologic TNM (T0-4 or N0, N1, N3, or M0), the degree of Clark level (Clark levels 1-2 or Clark levels 3-4), and tumor location (metastasis or regional cutaneous or regional lymph) subgroups, patients in the low-risk group had a longer survival duration compared to patients in the high-risk group, which suggested that our IAG signature was capable of predicting survival of patients in stratification analysis. (Supplement Figure 1 and Supplement Figure 2).

### 3.6. Comparison of the IAG Signature with Other Confirmed Melanoma Prognostic Biomarkers

**Figure 4:**
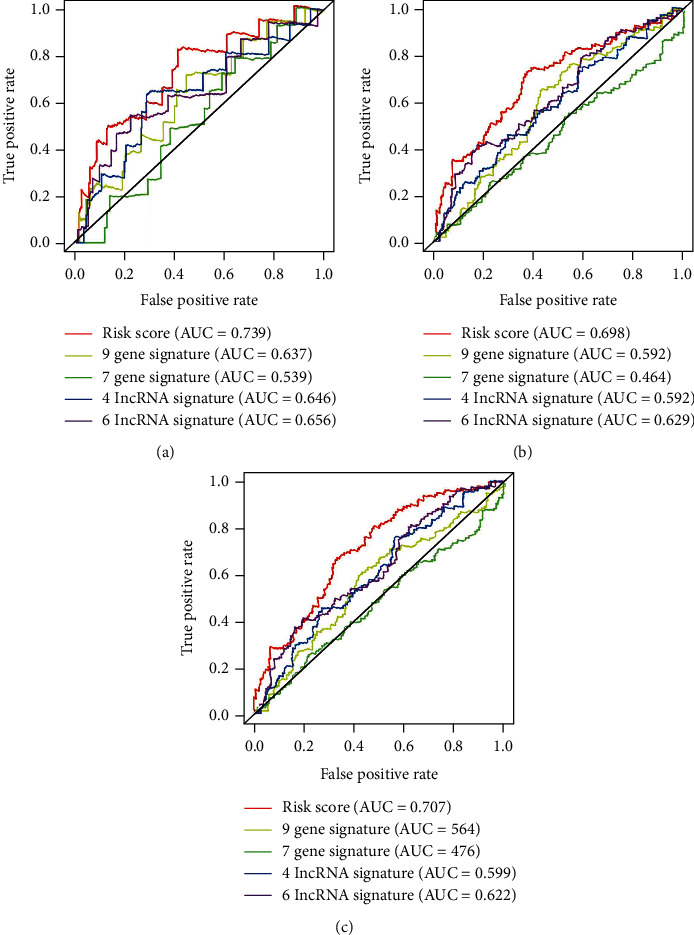
The AUC value for 1-year, 3-year, and 5-year OC of our six-IAG signature compared with other four gene-associated signatures.

Additionally, to identify whether the IAG signature had the ability of steady and dependable performance in the TCGA cohort, some melanoma prognostic predictors from other studies were selected for comparison [24–27]. The ROC curve analyses were performed, and the area under the ROC curve (AUC) was measured. Our IAG signature curves demonstrated the greatest AUC value (AUC = 0.739) compared with the 9-gene signature (AUC = 0.637), 7-gene signature (AUC = 0.539), 4-lncRNA signature (AUC = 0.646), and 6-lncRNA signature (AUC = 0.656) at an OS of 1 year. Similarly, the AUC values for 3-year and 5-year OC of the IAG signature were 0.698 and 0.707, respectively, which were higher than the values of 9-gene signature (3-year: AUC = 0.592, 5-year: AUC = 0.564), 7-gene signature (3-year: AUC = 0.464, 5-year: AUC = 0.476), 4-lncRNA signature (3-year: AUC = 0.592, 5-year: AUC = 0.599), and 6-lncRNA signature (3-year: AUC = 0.629, 5-year: AUC = 0.622) (Figure 4). These results underscored that our IAG signature was a top predictor for the prognosis assessment of melanoma and provided better stability, reliability, and veracity in predicting OS.

### 3.7. Connection of the Six IAG Signature with Immunotherapy-Related Biomarkers

**Figure 5:**
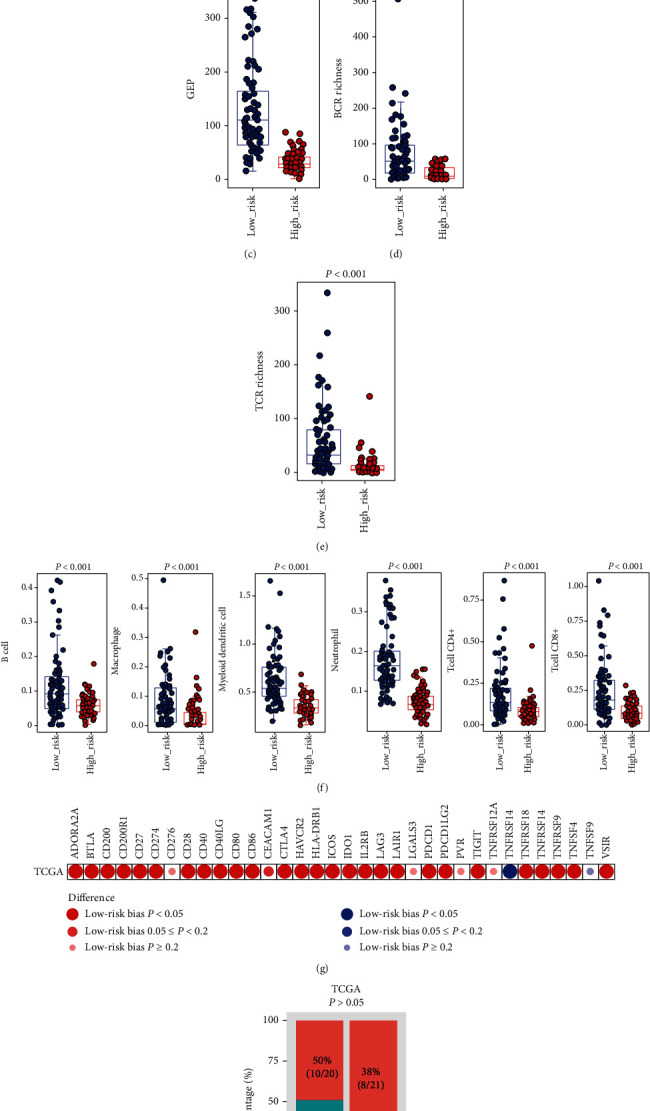
The relationship between the IAG signature and immunotherapy-related biomarkers. (a) The propensity score between the high-risk and low-risk groups. (b) CYT, (c) GEP, (d) BCR richness, and (e) TCR richness. (f) Six immune cell populations, including B cell, macrophage, myeloid dendritic cell, neutrophil, T cell CD4+, and T cell CD8+ between the high- and low-risk groups. (g) Differences in the mRNA expression level of 34 immune checkpoints. (h) Comparison of immunotherapy response rate between the high- and low-risk groups.

Recently, immunotherapy has become a promising clinical strategy to treat and even cure certain types of tumors, aiming at activating or promoting the activation of the immune system to attack cancer cells by natural mechanisms and improving antitumor immune responses with fewer off-target effects than other treatments to kill tumor cell [28–30]. More importantly, immune-checkpoint blockade (ICB) has provided a paradigm shift in the treatment of advanced-stage cancers and brought vital clinical benefits for melanoma patients [31, 32]. We explored the immune components analysis in the whole TCGA database. In order to reduce potential confounding effects, we utilized a propensity score algorithm, which was employed to reweight potential confounding effects, such as T stage, tumor stage, and Breslow depth in a multivariate manner. We visualized the propensity score distributions between the high-risk group and the low-risk group (Figure 5(a)). Then, we included all biomarkers used in patients with ICB treatment, as well as other significant biomarkers for immunotherapy, including immune cell populations, checkpoints, TCR/BCR, and aneuploidy score. Regarding ICB treatment data sets, we found that significantly higher CYT (*P* < 0.001; Figure 5(b)) and GEP (*P* < 0.001; Figure 5(c)) were in the low-risk group with melanoma. Consistent with the ICB treatment data sets, BCR/TCR richness scores (*P* < 0.001; Figures 5(d) and 5(e)) were higher in the low-risk group, too. Similarly, in terms of immune cell populations, B cell, macrophage, myeloid dendritic cell, neutrophil, T cell CD4+, and T cell CD8+ (all*P* < 0.001; Figure 5(f)) also demonstrated low-risk group biased. With respect to differences of the mRNA expression level of 34 immune checkpoints in melanoma, the results displayed that the expression levels of most immune checkpoints, such as ADORA2A, BTLA, CD200, CTLA4, CEACAM1, and PDCD1, were significantly higher in the low-risk group compared to the high-risk group (Figure 5(g)). In contrast, the expression level of TNFRSF14 showed the high-risk group biased. Further, we singled out the melanoma patients who were undergoing immunotherapy (*n* = 41), which contributed to different therapeutic results, including clinical progressive disease (PD), stable disease (SD), partial response (PR), and complete response (CR). After reasonable classification and evaluation, we divided patients into two subgroups (PD/SD and PR/CR) in the high-risk and low-risk groups, which represented different responses to immunotherapy. Unfortunately, the results demonstrated that there was no significant difference between the high- and low-risk groups in statistics that may be due to the small sample size (*P* > 0.05) (Figure 5(h)). In general, these findings demonstrated that our six IAG signature not only played a critical role in stratifying melanoma patients and predicting OS but also was significantly connected with ICB immunotherapy, BCR/TCR richness, immune cell populations, and immune checkpoints.

### 3.8. Verification of the Expression and Prognostic Value of IAGs in the Signature

The expression profiles of the six key IAGs between primary and metastatic melanoma tissue are presented in Supplement Figure 3. The result demonstrated that CCLB (*P* = 0.018), INHA (*P* < 0.001), and NDRG1 (*P* = 0.001) were significantly upregulated in metastatic melanoma, while LHB (*P* = 0.001) was significantly downregulated when compared with primary melanoma. However, the expression levels of IFITM1 (*P* = 0.643) and GBP2 (*P* = 0.320) were not significantly different between primary and metastatic melanoma tissues.

## 4. Discussion

Melanoma is the third most common skin cancer worldwide. But in fact, melanoma mortality remains highest among all types of skin cancer [33–35]. According to the latest cancer statistics reported, approximately 96,000 new cases of melanoma are diagnosed for 2019 in the United States, and more than 7000 patients eventually succumb to this tumor [36, 37]. Nevertheless, a variety of melanoma patients are treated with limited and similar therapies on account of lacking reliable and effective predictive tools to estimate patients' prognosis. Therefore, it is meaningful and necessary to identify accurate biomarkers to construct prognostic signatures for better predicting patients with refractory disease and worse survival and help to make decisions with regard to observation, surveillance, drug, surgery, and immunotherapy.

Recently, increasing evidence has illustrated that biomarkers based on tumor immunity can be applied for the diagnosis, prognosis, and treatment of tumor patients [38–40]. IAGs are now playing a critical role in tumor proliferation, differentiation, tumorigenesis, invasion, and metastasis in a variety of human cancers. During the last 5 years, no tumor has made an enormous breakthrough as melanoma in immunotherapy, but the efforts to uncover and explore mechanisms utilized by the tumor to avoid immune detection are still needed [15, 41, 42].

In the present study, under the Kaplan-Meier survival analyses, Cox regression analysis, LASSO regression analysis, we screened out six hub IAGs (INHA, NDRG1, IFITM1, LHB, GBP2, and CCL8). Further, we constructed and verified the six IAG prognostic signatures that could accurately stratify patients into the high- and low-risk subgroups with different survival results in the training and testing set. Among these IAGs, several studies displayed that these IAGs played an essential role in tumorigenesis and development, even in melanoma. For instance, INHA was defined as a clinically key target in treating people with tuberculosis [43, 44]. NDRG1 played an indispensable role in myelination of the peripheral nervous system and was linked with the increasing rate of metastasis and death [45, 46]. IFITM1 was reported to participate in the signals of cell adhesion and antiproliferation transduction [47]. And the overexpression of IFITM1 was confirmed to be correlated with the progressing of several cancers including ovarian cancer, breast cancer, oral cancer, lymphoma, and leukemia [48]. GBP2 was found as a TGF-b target gene induced in metastatic breast cancer cells [49]. CCL8 may enhance the ability of metastasis formation in melanomas as a chemoattractant. The local CCL8-rich environment could promote the selection of tumor cells with metastatic capacity, and the high CCL8 concentration inhibited the migration of tumor cells [50]. There are few relevant works of literature reported involving in LHB, and the further research is needed. As a consequence, studies as mentioned above revealed the reasonability and accuracy of our six IAG signatures in cancer initiation and progression.

The KM survival curves showed that high risk scores were related to poor OS in both the training set and the testing set. Next, the time-dependent ROC analysis was performed, and the results proved that our prognostic signature had better sensitivity and specificity in predicting melanoma patients' survival at 1-year, 3-year, and 5-year OC. Using the univariate and multivariate Cox regression models, the IAG signature was an independent prognostic factor in melanoma patients. Furthermore, melanoma patients were further stratified into several subgroups which were distributed into the high-risk and low-risk groups.

Moreover, it gave a clear illustration that in the whole TCGA database, the six-IAG signature exceeded other melanoma prognostic predictors, including a nine-gene signature, the seven-marker signature, the four-lncRNA prognostic signature, and the six-lncRNA signature [24]. The ROC curve analyses demonstrated that our signature provided better performance in predicting OS than other known biomarkers. Meanwhile, it was of great importance to identify the reliability and veracity of our IAG signature when further melanoma patients were available.

As for the treatments of melanoma, over the past decades, the management of melanoma has rapidly developed with the introduction of novel drug classes, such as BRAF and MEK pathway-targeted inhibitors and targeting immune check-point inhibitors consisting of CTLA-4, PD-1, PD-L1, and PD-L2 [32, 36, 51, 52]. Dramatic progression in survival outcomes has been revealed; with the therapy of immune checkpoint blockade (ICB), the melanoma patients' median overall survival was more than 30 months [53, 54]. In our study, we explored the relationship between the risk score and immunotherapy-related biomarkers. The results showed that the IAG signature was significantly connected with ICB immunotherapy, BCR/TCR richness, immune cell populations, and immune checkpoints.

The strength of our study was that it was the first time for us to carry out the systematic analysis of immune-related genes in melanoma, and our IAG-based signature was successfully created and accurately estimated in the testing set. However, several limitations should be mentioned. Firstly, the research was retrospective, and we only enrolled in the TCGA database to construct and validate the IAG signature. Therefore, the accuracy and availability of our biomarker remained to be tested in other datasets, even in the prospective studies. Secondly, concerning the ICB immunotherapy, we might need more samples and more complete clinicopathological data to identify the robust relationship between our IAG signature and the immunotherapy. Finally, it was of great importance to conduct several functional experiments to clarify the roles of the six IAGs in melanoma. Despite the deficiencies mentioned above, the prognostic value of the IAG signature for OS in melanoma patients could not be ignored. In the future, multi-institutional and well-designed researches are needed to verify our findings.

## 5. Conclusion

In conclusion, we identified six immune-associated gene comprising of INHA, NDRG1, IFITM1, LHB, GBP2, and CCL8 to establish the risk score. Meanwhile, our six-IAG signature was certified to predict patients' overall survival and associated with immunotherapy-related biomarkers. However, further prospective studies and clinical trials are required to validate the efficiency, accuracy, and value of this signature and offer a panoramic view of the immune mechanisms in melanoma.

## Figures and Tables

**Table 1:** Clinicopathological characteristics of melanoma patients from TCGA database.

Characteristics		Training cohort (*N* = 289)		Validation cohort (*N* = 145)		
	Groups	No.	%	No.	%	*P* value
Gender	Male	185	64.0	89	61.4	0.592
	Female	104	36.0	56	38.6	
Age at diagnosis	<50	89	30.8	44	30.4	0.898
	50-70	132	45.7	64	44.1	
	>70	68	23.5	37	25.5	
Race	White	274	94.8	138	95.2	0.871
	Others/unknown	15	5.2	7	4.8	
Breslow depth	<2	80	27.7	46	31.7	0.658
	2-5	79	27.3	34	23.4	
	>5	59	20.4	33	22.8	
	Unknown	71	24.6	32	22.1	
Clark level	I-II	17	5.9	6	4.1	0.491
	III-IV	151	52.2	80	55.2	
	V	28	9.7	19	13.1	
	Unknown	93	32.2	40	27.6	
Pathologic T	T0-T2	82	28.4	57	39.3	0.342
	T3-T4	149	51.5	68	46.9	
	Unknown	58	20.1	20	13.8	
Pathologic N	N0	142	49.1	74	51.0	0.719
	N1	48	16.6	23	15.9	
	N2	28	9.7	19	13.1	
	N3	37	12.8	16	11.0	
	Unknown	34	11.8	13	9.0	
Pathologic M	M0	257	88.9	128	88.3	0.476
	M1	17	5.9	6	4.1	
	Unknown	15	5.2	11	7.6	
Pathologic stage	0	5	1.8	1	0.7	0.651
	I	46	15.9	30	20.7	
	II	80	27.7	41	28.3	
	III	109	37.7	55	37.9	
	IV	16	5.5	6	4.1	
	Unknown	33	11.4	12	8.3	
Tumor location	Primary tumor	52	18.0	26	17.9	0.592
	Regional cutaneous or Subcutaneous tissue	46	15.9	27	18.6	
	Regional lymph node	142	49.2	74	51.1	
	Distant metastasis	46	15.9	18	12.4	
	Unknown	3	1.0	0	0	
Anatomic site	Extremities	129	44.6	58	40.0	0.792
	Head and neck	20	6.9	12	8.3	
	Trunk	100	34.6	52	35.9	
	Others/unknown	40	13.9	23	15.8	

## Data Availability

RNA-sequencing (RNA-seq) expression profile and related clinical follow-up parameters of melanoma cohorts were downloaded from The Cancer Genome Atlas (TCGA) RNA-seq (TCGA, http://cancergenome.nih.gov/). The comprehensive list of immune related-genes was extracted from The Immunology Database and Analysis Portal (ImmPort, https://immport.niaid.nih.gov/).

## Supplementary Materials

Supplementary 1 Supplement Figure 1: Kaplan-Meier analysis of the overall survival melanoma patients by different prognostic-associated factors (anatomic site, tumor location, Breslow depth, and Clark level).

Supplementary 2 Supplement Figure 2: Kaplan-Meier analysis of the overall survival melanoma patients by different prognostic-associated factors (T0-2, T3-4, N0, N1, N3, and M0).

Supplementary 3 Supplement Figure 3: the expression profiles of the six key IAGs between the primary and metastatic melanoma tissues.
