# Supplementary material for: The Identification and Validation of a Robust Immune-Associated Gene Signature in Cutaneous Melanoma
Source: J Immunol Res. 2021 Feb 19;2021:6686284. doi: 10.1155/2021/6686284 (PMC7911606; doi:10.1155/2021/6686284)
Supplement: Supplementary 1 — Supplement Figure 1: Kaplan-Meier analysis of the overall survival melanoma patients by different prognostic-associated factors (anatomic site, tumor location, Breslow depth, and Clark level). [file 6686284.f1.pdf]

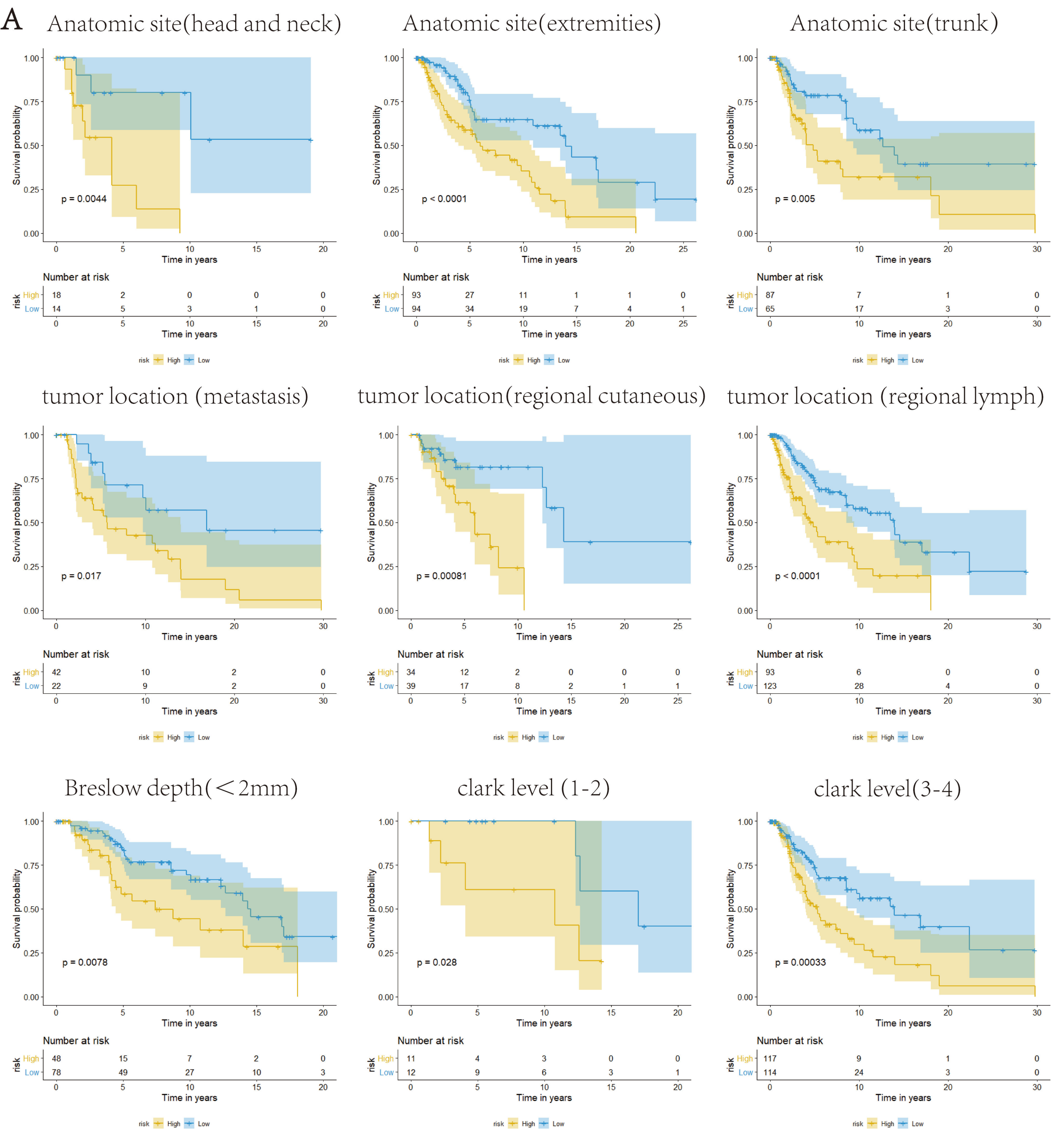
A
Anatomic site(head and neck)
p = 0.0044
Number at risk
High 18 2 0 0 0
Low 14 5 3 1 0
Anatomic site(extremities)
p < 0.0001
Number at risk
High 93 27 11 1 1 0
Low 94 34 19 7 4 1
Anatomic site(trunk)
p = 0.005
Number at risk
High 87 7 1 0
Low 65 17 3 0
tumor location (metastasis)
p = 0.017
Number at risk
High 42 10 2 0
Low 22 9 2 0
tumor location(regional cutaneous)
p = 0.00081
Number at risk
High 34 12 2 0 0 0
Low 39 17 8 2 1 1
tumor location (regional lymph)
p < 0.0001
Number at risk
High 93 6 0 0
Low 123 28 4 0
Breslow depth(＜2mm)
p = 0.0078
Number at risk
High 48 15 7 2 0
Low 78 49 27 10 3
clark level (1-2)
p = 0.028
Number at risk
High 11 4 3 0 0
Low 12 9 6 3 1
clark level(3-4)
p = 0.00033
Number at risk
High 117 9 1 0
Low 114 24 3 0
Survival probability
Time in years
risk
High
Low
