# Supplementary figures and images for: The Identification and Validation of a Robust Immune-Associated Gene Signature in Cutaneous Melanoma
Source: J Immunol Res. 2021 Feb 19;2021:6686284. doi: 10.1155/2021/6686284 (PMC7911606; doi:10.1155/2021/6686284)

B

T0-T2

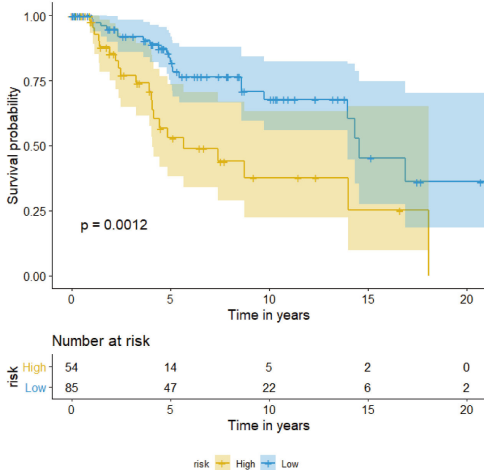

T3-T4

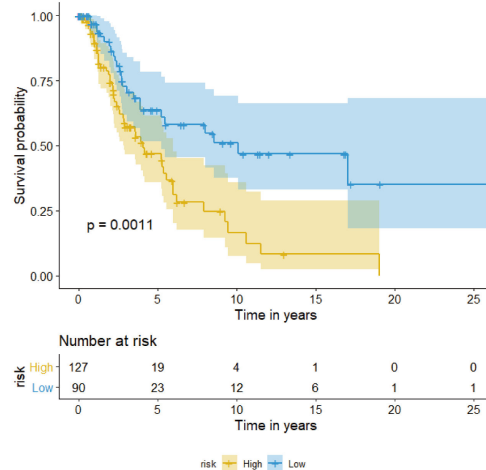

N0

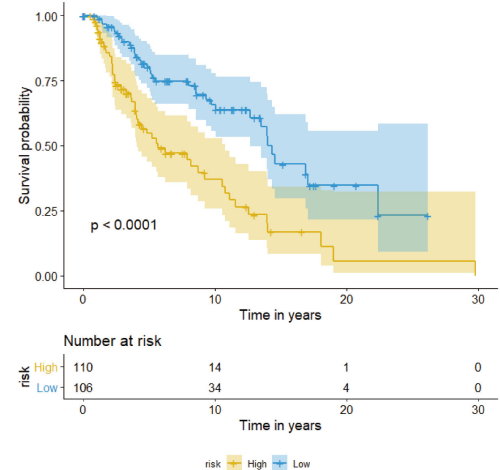

N1

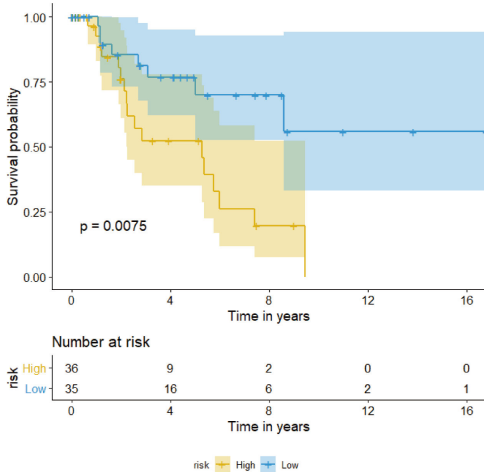

N3

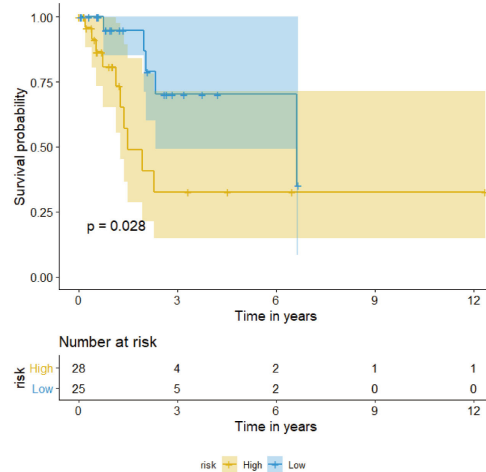

M0

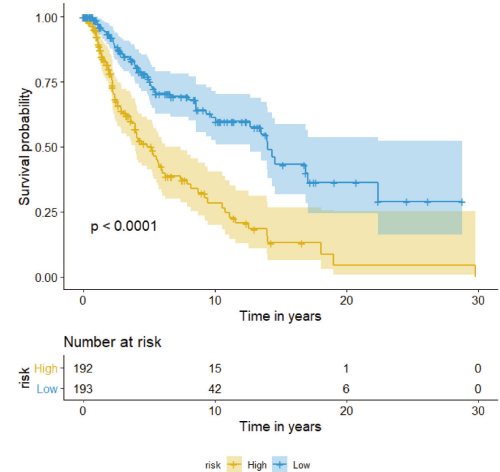

Supplement: Supplementary 2 — Supplement Figure 2: Kaplan-Meier analysis of the overall survival melanoma patients by different prognostic-associated factors (T0-2, T3-4, N0, N1, N3, and M0). [file 6686284.f2.pdf]

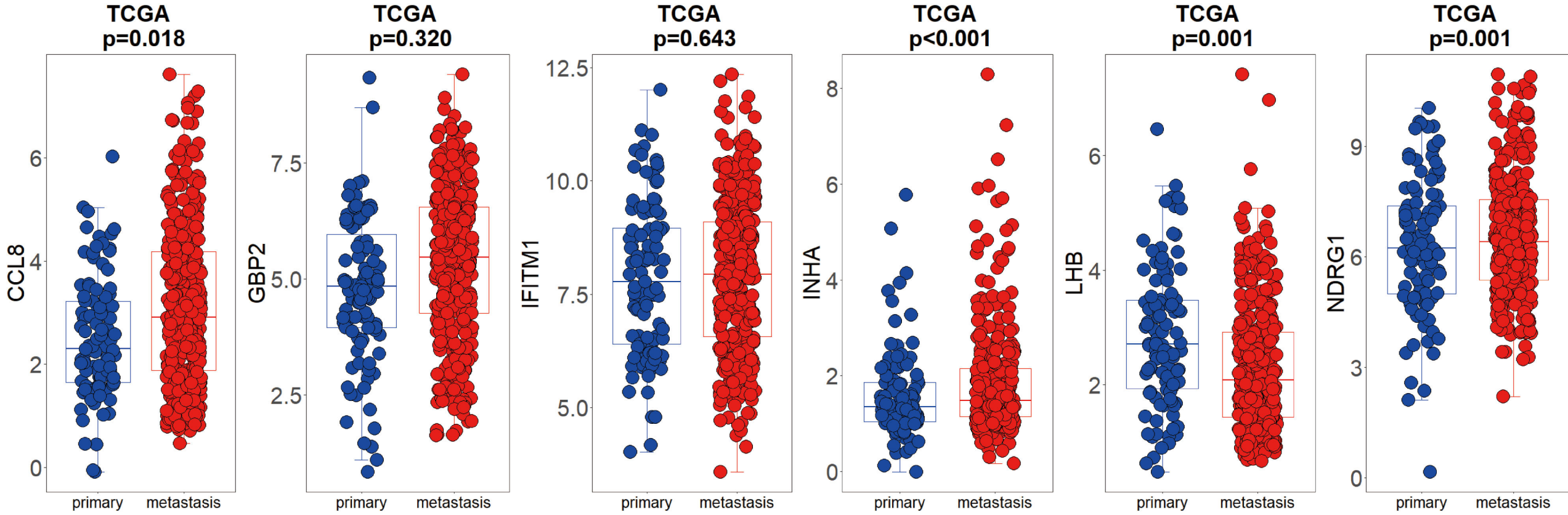

Supplement: Supplementary 3 — Supplement Figure 3: the expression profiles of the six key IAGs between the primary and metastatic melanoma tissues. [file 6686284.f3.pdf]
